# Proteomic analysis of *Nrk* gene-disrupted placental tissue cells explains physiological significance of NRK

**DOI:** 10.1186/s13104-019-4818-7

**Published:** 2019-11-29

**Authors:** Kimitoshi Denda, Kanako Ida, Masataka Tanno, Kanako Nakao-Wakabayashi, Masayuki Komada, Nobuhiro Hayashi

**Affiliations:** 1 School of Life Science and Technology, Tokyo Institute of Technology, (M6-6) 2-12-1 Ookayama, Meguro-ku, Tokyo, 152-8550 Japan; 2 Department of Pathology, Tokyo Nishi Tokushukai Hospital, Akishima, Japan; 3 Cell Biology Unit, Institute of Innovative Research, Tokyo Institute of Technology, Yokohama, Japan

**Keywords:** Placentomegaly, Dystocia, Breast tumor, Placenta, Protein kinase

## Abstract

**Objective:**

NRK is a unique X chromosome-linked protein kinase expressed predominantly in placenta. The gene knockout causes placental overgrowth and delayed labor of *Nrk*-null fetuses from dams in mouse. To clarify unknown mechanisms behind the *Nrk*-null phenotypes, protein expression profiles were analyzed in the *Nrk*-null placenta using a high-performance two-dimensional electrophoresis methodology.

**Results:**

Among around 1800 spots detected, we characterized a dozen protein spots whose expression levels were significantly altered in the *Nrk*-null placenta compared to wild-type. Analyzing these data sets is expected to reflect the difference physiologically in the presence or absence of NRK, facilitating the development of therapeutic strategies.

## Introduction

Parturition essential for the survival and proliferation of eutherian mammals is considered to be strictly regulated for ensuring the safety of the next-generation descendants [1, 2]. During pregnancy, the fetus and placenta are most likely to communicate with each other until delivery in seeking the safe and secure opportunity for birth. However, little is known about the shared mechanism that controls communication between the mother and the unborn. By analyzing various single-gene knockout (KO) mice, several candidates of the messaging molecules involved in the negotiation for selecting the best timing of birth have been identified to date [3–9]. One of the most prominent of these candidates is NRK [10].

NRK (NIK-related kinase), highly expressed in the placenta, is a physiologically unique X-encoded Ser/Thr protein kinase [10–12]. We previously reported that the *Nrk* gene KO causes placental overgrowth, indicating that NRK is a crucial modulator of cell proliferation and development in placental tissues [10]. Furthermore, the *Nrk*-null fetuses influence the pregnant dam to delay delivery. Together with subsequent work using intrauterine embryonic transfer of *Nrk*-null fetuses into wild-type (WT) dams [10], these results suggested that NRK is required for mediating one or more unidentified delivery-inducing signals dispatched from the placenta. In addition, we have found that *Nrk* mutant female mice develop breast tumors frequently, suggesting that NRK is a tumor-suppressor gene [13].

These results tempted us to clarify the molecular mechanisms behind the *Nrk*-null phenotypes by using proteomic analysis to profile the protein expression of key regulators in the placenta of *Nrk* KO fetuses. We presented herein detailed two-dimensional electrophoresis (2DE) reference maps of the mutant mouse placenta to establish a NRK-connected placental database, available worldwide, that contains information on protein species identified by 2DE.

## Main text

### Experimental methods

We applied a high-performance 2DE methodology to analyse mouse placenta harvested in the third trimester of pregnancy. Variations in protein expression levels were defined by comparing individual protein spots on the resulting gels. The trophoblast tissue layer samples were subsequently dissected from the whole placenta as in Additional file 1: Figure S1. After treating the samples using a 2-D Clean-Up Kit (GE Healthcare Ltd., UK), protein quantification, isoelectric focusing of proteins, 2DE, gel-staining, and data analysis were performed as described by Wong et al. [14].

### Results and discussion

**Fig. 1:**
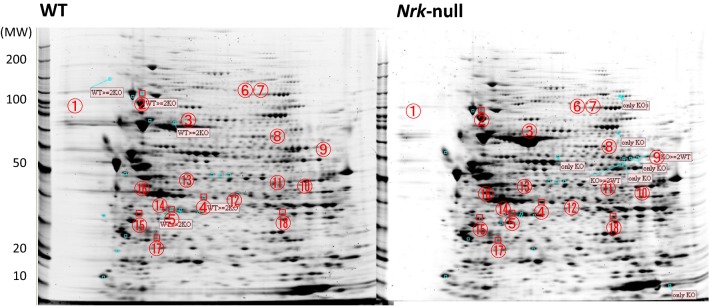
2DE map of the *Nrk*^−/−^ mouse placenta. Representative 2DE protein profiles with the protein spots marked as differentially regulated on *Nrk*^−/−^ placentas at embryonic day 18.5 (E18.5). We performed proteomics in over triplicate experiments and presented herein the dataset for late-pregnancy placental tissues disrupted for the tumor suppressor gene *Nrk*. Pairs of WT/KO gel images were compared to identify 18 protein spots (from approximately 1800 detected spots) that differed significantly in the 2DE images. The identities of the spots, as determined by LC–ESI–MS/MS, are presented in Table 1. Total protein fractions were separated by isoelectric focusing on a Multiphor II system (GE Healthcare Ltd., UK) and SDS-PAGE using a NuPAGE 4–12% Bis–Tris Z00m Gel (Thermo Fisher Scientific). SYPRO Ruby-stained gels were scanned using the Typhoon Imaging System (GE Healthcare Ltd., UK) and analyzed using Image Master 2D Platinum 7.0 software (GE Healthcare Ltd., UK). Spots corresponding to differentially expressed proteins are labeled with numbers

In Table 1, we calculated the individual average from six independent trials of 2DE (Fig. 1). Although some variations are observed, it is considered that the increase or decrease in protein amount of each spot has been verified. Among several identified proteins whose levels were attenuated in the mutant, annexin A3 and A5 belong to the annexin family composed of functionally diverged Ca^2+^-dependent membrane phospholipid-bound intracellular proteins [15]. Downregulation of annexin A3 (Spot 5) inhibits growth, migration, invasion, and metastasis of lung cancer cells by suppressing the MEK/ERK signaling pathway [16]. Annexin A3 may be involved in the metabolism of estrogens; this function could be relevant, given that estrogen-induced disruption of the intracellular microenvironment leads to membrane damage and cell cycle arrest [17]. Annexin A5 (Spot 15) is known to be an expedient diagnostic marker for detecting apoptotic cells that is a component of the outer leaflet of the plasma membrane [18]. Decreased levels of Annexin A5 in the *Nrk* KO placenta tempts us to speculate that NRK functions by potentiating cell death, thereby promoting excess proliferation of specific tissue layers without NRK activity, and leading to phenotypes such as placentomegaly and breast tumorigenesis.

**Table 1:** List of mouse placental proteins whose expression differed significantly between control and *Nrk*^−/−^ 2DE performed on proteins from E18.5 concepti, as identified by LC-MS/MS analysis

Spot Nr	Protein description	Protein entry	Accession	Score	avgMass	seqCover (%)	Effect size^a^	SD	*t* test (n = 6)
1	Protein 2210010C04Rik OS Mus musculus GN 2210010C04Rik PE 2 SV 1	Q9CPN9_MOUSE	Q9CPN9	435	26422	8.10	− 0.710	0.653	0.009
2	Heat shock protein HSP 90 beta OS Mus musculus GN Hsp90ab1 PE 1 SV 3	HS90B_MOUSE	P11499	102	83281	3.59	− 0.248	0.126	0.020
3	Stress 70 protein mitochondrial OS Mus musculus GN Hspa9 PE 1 SV 3	GRP75_MOUSE	P38647	1030	73461	39.62	− 0.018	0.774	0.942
4	Protein Serpinb9f OS Mus musculus GN Serpinb9f PE 2 SV 1	Q80UK5_MOUSE	Q80UK5	535	43034	16.98	− 0.126	0.096	0.148
5	Annexin A3 OS Mus musculus GN Anxa3 PE 1 SV 4	ANXA3_MOUSE	O35639	556	36384	27.24	0.127	0.169	0.062
6	Staphylococcal nuclease domain containing protein 1 OS Mus musculus GN Snd1 PE 1 SV 1	SND1_MOUSE	Q78PY7	50	102,088	4.95	0.109	0.682	0.846
7^b^	–	–	–	–	–	–	− 0.285	0.555	0.492
8	Protein 2210010C04Rik OS Mus musculus GN 2210010C04Rik PE 2 SV 1	Q9CPN9_MOUSE	Q9CPN9	727	26422	4.86	0.309	0.233	0.031
9	Pyruvate kinase isozymes M1 M2 OS Mus musculus GN Pkm PE 1 SV 4	KPYM_MOUSE	P52480	3716	57845	50.66	− 0.272	0.498	0.294
10^b^	–	–	–	–	–	–	0.221	0.347	0.198
11	Protein disulfide isomerase A5 OS Mus musculus GN Pdia5 PE 2 SV 1	PDIA5_MOUSE	Q921X9	302	59267	15.67	0.666	0.816	0.116
12	Glutamate dehydrogenase 1 mitochondrial OS Mus musculus GN Glud1 PE 1 SV 1	DHE3_MOUSE	P26443	176	61337	10.75	0.021	0.539	0.654
13	T complex protein 1 subunit alpha OS Mus musculus GN Tcp1 PE 1 SV 3	TCPA_MOUSE	P11983	465	60449	30.22	0.217	0.271	0.208
14	40S ribosomal protein SA OS Mus musculus GN Rpsa PE 1 SV 4	RSSA_MOUSE	P14206	3256	32838	47.46	0.110	0.148	0.115
15	Annexin A5 OS Mus musculus GN Anxa5 PE 1 SV 1	ANXA5_MOUSE	P48036	12996	35753	83.39	− 0.114	0.179	0.223
16	SPARC OS Mus musculus GN Sparc PE 1 SV 1	SPRC_MOUSE	P07214	126	34450	11.59	− 0.256	0.597	0.235
17	Calpain small subunit 1 OS Mus musculus GN Capns1 PE 2 SV 1	CPNS1_MOUSE	O88456	951	28463	31.97	− 0.068	0.303	0.531
18	Annexin A2 OS Mus musculus GN Anxa2 PE 1 SV 2	ANXA2_MOUSE	P07356	14770	38676	67.26	0.102	0.410	0.509

Specified proteins of the *Nrk*^−/−^ mouse placenta are indicated

^a^Individual effect size is an average calculated from six independent trials

^b^The protein spots corresponding spot number 7 and 10 could not be specified

Calpain, a calcium-regulated cysteine protease corresponded to Spot 17, the intensity of which was decreased in the *Nrk* KO placenta. Calpain is implicated in cytoskeletal remodeling and signal transduction. Calpain-mediated proteolytic cleavage induces cytoskeletal dynamics. This activity is altered during aging and in the progression of numerous diseases, including calcium-dependent disorders and pathological conditions [19, 20]. Spot 2, a signal decreased in the KO placenta, was identified as HSP90, a molecular chaperone with numerous client proteins. Many HSP90 inhibitors are undergoing study for potential use as clinical therapies [21]. Calpain is known to regulate HSP90 expression by cleaving HSP90 directly. On the other hand, HSP90 has been reported to affect the activity of calpain, suggesting that interactions between HSP90 and calpain jointly contribute to physiological functions. Indeed, genetic disruption of the calpain-encoding gene or treatment with HSP90 inhibitors has been shown to yield attenuation of mammary tumorigenesis [22].

Spot 4, a signal decreased in the KO placenta, corresponded to serpins of clade B (serpinb9f), a unique class of intracellular protease inhibitors [23]. Among these inhibitors, serpin B9 is a well-studied specific inhibitor of granzyme B [24]. It seems likely that the granzyme-mediated proteolysis is important for the immune response to infection or tumorigenesis. Changes in serpin levels are expected to cause cell damage in normal tissues. Therefore, NRK may contribute to a cytoprotective function by safeguarding lymphocytes from granzymes.

For the purposes of the present study, proteomics may be a more informative approach than gene expression (transcriptional) profiling, given that transcript accumulation does not always correlate with qualitative or quantitative differences in protein levels and often fails to reflect in vivo protein localization, depending on the tissue. Our data tempted to speculate that dysfunction of NRK leads to defects in cellular proliferation, cell cycle progression, resistance to apoptosis, and oncogenesis. Also, recent progress in advanced mass spectrometry methods is expected to enable us to monitor numerous phosphorylation sites in proteins. Together with the genomic discoveries through genome-wide association studies reported recently [25], further profiling analyses of the gene product changes in the *Nrk*-gene-mutants is expected to clarify the functional mechanism of fetoplacental development and differentiation during pregnancy, facilitating the identification of potential targets of current chemotherapeutic treatments available for perinatal medicine.

### Limitations

The main limitation of our research was that we couldn’t verify why each identified expressed protein decreased in NRK-deficient placental tissue cells which could explain how NRK works physiologically in the state of health. Elucidating the physiological role of NRK in future studies would not only become one target protein for drug discovery but also helps to improve human health.

## Supplementary information

**Additional file 1: Figure S1.** Dissection procedure for collecting layer-enriched tissue samples from the mouse whole placenta in late gestation.

## Data Availability

All data generated or analyzed during this study are included in this published article and its supplementary information files.

## Abbreviations

2DE: two-dimensional gel electrophoresis
dpc: days postcoitum
ER: estrogen receptor
H&E: hematoxylin and eosin
LC–MS/MS: liquid chromatography-tandem mass spectrometry
PCR: polymerase chain reaction
WT: wild-type
KO: knockout
