# Supplementary figures and images for: Proteomic analysis of Nrk gene-disrupted placental tissue cells explains physiological significance of NRK
Source: BMC Res Notes. 2019 Nov 29;12:785. doi: 10.1186/s13104-019-4818-7 (PMC6884884; doi:10.1186/s13104-019-4818-7)

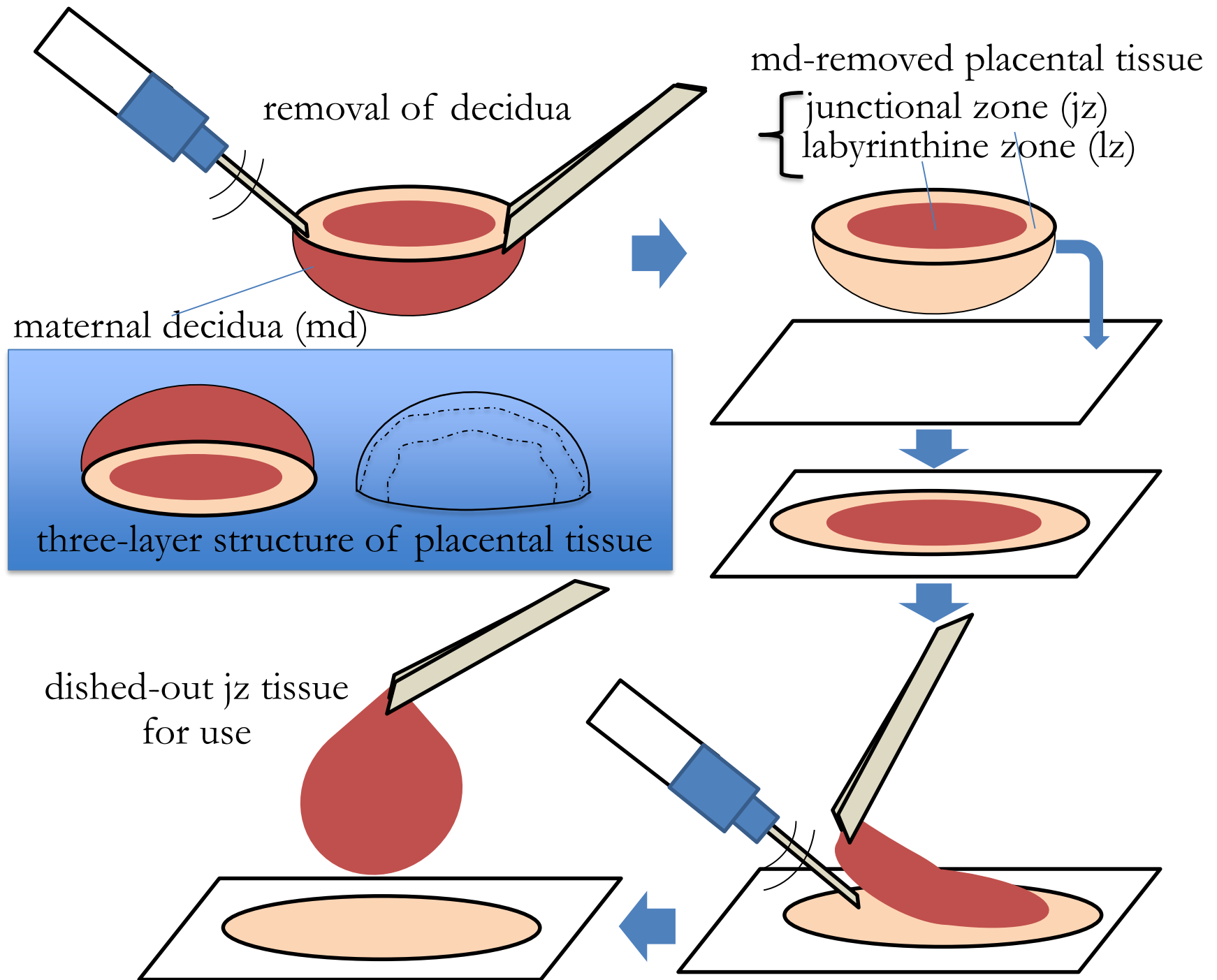

Supplement: Supplementary file 1 — Additional file 1: Figure S1. Dissection procedure for collecting layer-enriched tissue samples from the mouse whole placenta in late gestation. [file 13104_2019_4818_MOESM1_ESM.pdf]
